# Blood brain barrier leakage is not a consistent feature of white matter lesions in CADASIL

**DOI:** 10.1186/s40478-019-0844-x

**Published:** 2019-11-21

**Authors:** Rikesh M. Rajani, Julien Ratelade, Valérie Domenga-Denier, Yoshiki Hase, Hannu Kalimo, Raj N. Kalaria, Anne Joutel

**Affiliations:** 1 Institute of Psychiatry and Neuroscience of Paris – INSERM UMR1266, Paris Descartes University, 102-108 Rue de la Santé, 75014 Paris, France; 2 Neurovascular Research Group, Institute of Neuroscience, Newcastle University, Campus for Ageing and Vitality, Newcastle upon Tyne, NE4 5PL UK; 3 Department of Pathology, Haartman Institute, University of Helsinki, FIN-00014 Helsinki, Finland

**Keywords:** CADASIL, Small vessel disease, Blood brain barrier, White matter lesions, Pericytes

## Abstract

Cerebral autosomal dominant arteriopathy with subcortical infarcts and leukoencephalopathy (CADASIL) is a genetic paradigm of small vessel disease (SVD) caused by *NOTCH3* mutations that stereotypically lead to the vascular accumulation of NOTCH3 around smooth muscle cells and pericytes. White matter (WM) lesions (WMLs) are the earliest and most frequent abnormalities, and can be associated with lacunar infarcts and enlarged perivascular spaces (ePVS). The prevailing view is that blood brain barrier (BBB) leakage, possibly mediated by pericyte deficiency, plays a pivotal role in the formation of WMLs. Herein, we investigated the involvement of BBB leakage and pericyte loss in CADASIL WMLs. Using post-mortem brain tissue from 12 CADASIL patients and 10 age-matched controls, we found that WMLs are heterogeneous, and that BBB leakage reflects the heterogeneity. Specifically, while fibrinogen extravasation was significantly increased in WMLs surrounding ePVS and lacunes, levels of fibrinogen leakage were comparable in WMLs without other pathology (“pure” WMLs) to those seen in the normal appearing WM of patients and controls. In a mouse model of CADASIL, which develops WMLs but no lacunes or ePVS, we detected no extravasation of endogenous fibrinogen, nor of injected small or large tracers in WMLs. Moreover, there was no evidence of pericyte coverage modification in any type of WML in either CADASIL patients or mice. These data together indicate that WMLs in CADASIL encompass distinct classes of WM changes and argue against the prevailing hypothesis that pericyte coverage loss and BBB leakage are the primary drivers of WMLs. Our results also have important implications for the interpretation of studies on the BBB in living patients, which may misinterpret evidence of BBB leakage within WM hyperintensities as suggesting a BBB related mechanism for all WMLs, when in fact this may only apply to a subset of these lesions.

## Introduction

Cerebral small vessel disease (SVD) is the leading cause of vascular dementia and a major contributor to stroke and disability [37]. Cerebral autosomal dominant arteriopathy with subcortical infarcts and leukoencephalopathy (CADASIL) is the most common monogenic form of SVD, affecting 2–5 in 100,000 people [43]. It is caused by mutations in the *NOTCH3* gene which lead to the characteristic and early accumulation of the NOTCH3 extracellular domain (Notch3^ECD^) around smooth muscle cells and pericytes in patients and mice with CADASIL [8, 25–27, 31, 45, 50].

CADASIL is a genetic paradigm of SVD which presents with all clinical and neuroimaging features of sporadic SVD [6]. CADASIL resembles sporadic SVD to such an extent that it has been used as a model in trials of drugs to treat vascular dementia [16]. One of the hallmarks of SVD is the presence of white matter hyperintensities (WMH) on magnetic resonance imaging (MRI) of the brain. WMH are the earliest and most frequent MRI abnormalities in CADASIL patients, appearing as punctiform lesions from around 30 years of age and becoming more diffuse and symmetrical with age. While predominating in the periventricular areas and the centrum semiovale, WMH occur at three locations which are highly suggestive of CADASIL: the anterior part of the temporal lobes, the external capsules, and the superior frontal gyri [6]. WMH on MRI are associated with cognitive decline in both the general population and high risk groups, though brain atrophy is a stronger determinant of clinical impairment in CADASIL patients [14, 47].

Another defining pathology of SVD is the presence of lacunes, fluid-filled cavities presumed to be the healed stages of deep brain infarcts [48]. These can be found throughout the white matter (WM) and the deep grey matter (GM) in the brain. In CADASIL, lacunes essentially occur within the same area as WM lesions, but at a later age than WM lesions [6]. Enlarged perivascular spaces (ePVS) have recently been recognised as another key feature of SVD on MRI and are thought to correspond to enlarged spaces around arteries and arterioles allowing freer movement of water [48]. The resolution limits of conventional MRI sequences mean that it is predominantly larger ePVS (> 1 mm diameter) around penetrating arteries which are easily visible, and the profile of following a vessel is key to distinguishing these from smaller lacunes [48]. A high density of ePVS is also seen in the basal ganglia, in a state termed “état criblé”. CADASIL patients show ePVS with a similar distribution, as well as a high density of ePVS in the temporal lobe [11].

The mechanisms by which white matter lesions (WMLs) form in SVD, and CADASIL in particular, remain elusive, however it has been suggested that a loss of integrity of the blood brain barrier (BBB) is pivotal in their pathophysiology [24, 38]. The BBB is the structure which prevents substances from the blood entering the brain parenchyma [1]. It is formed by tight junctions (TJs) between brain endothelial cells, and its function is supported by pericytes, the mural cells around capillaries. Notably, a loss of pericytes in the mouse increases the permeability of the BBB to water and a range of low and high molecular weight molecules [2, 12]. Investigations into the integrity of the BBB in sporadic SVD have shown mixed results, with some MRI and biochemical studies suggesting an increase in BBB permeability [18], while other histopathological studies have suggested that this is not the case [4]. This discrepancy may reflect differences in sensitivity or reliability of the different techniques used. The possibility that WMLs in SVD might be caused by an increase in BBB permeability has been bolstered by recent work in pericyte deficient mice suggesting a pathological link between BBB breakdown and WM pathology; it has been suggested that in this model fibrinogen toxicity can cause oligodendrocyte death and WM damage [32]. However, the integrity of the BBB has not been studied in the WM in CADASIL.

Here, we tested the hypothesis that pericyte loss and subsequent BBB breakdown may cause WMLs in CADASIL. To achieve this, we studied post-mortem brains from CADASIL patients that represent the whole spectrum of the disease. We also used a well-established mouse model of CADASIL, allowing us to perform in vivo studies and study an early stage of the disease process.

## Methods

### Human brain tissue

Ethical approval for the use of human tissue was provided by the INSERM Institutional Review Board (IRB00003888) to AJ. Tissue was collected in line with local guidelines. Post-mortem human brain tissue from the frontal lobe (with two exceptions, see Additional file 1: Tables S1-S2) was analysed in two cohorts. The first cohort included eight CADASIL patients and six controls with no neurological disease, with samples from Paris (INSERM and GIE-NeuroCeb brain bank), Turku University and the University of Edinburgh. These samples were provided as paraffin embedded blocks, and sectioned at 5 μm onto standard microscope slides (25 mm × 75 mm). The controls and patients in this cohort were 50 and 50% male respectively; 60.8 ± 14.7 (mean ± standard deviation) and 62.9 ± 9.2 years old respectively; and had a post-mortem delay of 18.6 ± 9.4 and 16.9 ± 8.9 h respectively. The second cohort included four CADASIL patients and four controls with samples provided by Newcastle University as 10 μm thick paraffin sections on large microscope slides (50 mm × 75 mm). The controls and patients in this cohort were 25 and 50% male respectively; 62.5 ± 10.5 and 60.8 ± 8.5 years old respectively; and had a post-mortem delay of 39.5 ± 11.8 and 21.3 ± 6.2 h respectively. Full details of the human controls and patients can be found in Additional file 1: Tables S1-S2.

### Animals

Animal experiments were conducted in full accordance with the guidelines of our local institutional Animal Care and Use Committee (Lariboisière-Villemin, CEA9). Tg*Notch3*^WT^ mice and Tg*Notch3*^R169C^ mice were maintained on an FVB/N background and overexpress the rat *Notch3* gene, either in the wild type form or containing the mutation R169C found in CADASIL patients respectively [27]. These mice were used at ages between 14 and 16 months. *Col4a1*^+/G498V^ mice were maintained on a C57BL/6 background and bear a mutation in the *Col4a1* gene which is found in HANAC syndrome patients [7]. HANAC syndrome is another genetic form of SVD, which is part of Collagen type IV related SVD, and mice bearing this mutation have previously been shown to display a transient defect of the BBB at 1 month of age which disappears with age [42]. These mice were used at 1 month of age.

### Injection of tracers

Mice were injected by tail vein injection with either 100 μl of cadaverine-AF555 (0.5 mg/ml; ThermoFisher; A30677) or 200 μl of a combination of a 70 kDa biotinylated dextran (12.5 mg/ml; ThermoFisher; D1857) and albumin-AF488 (5 mg/ml; ThermoFisher; A34781). *Col4a1*^+/G498V^ mice (cadaverine) or drill injury mice (dextran and albumin) served as positive controls. Cadaverine was used as its small molecular weight (950 Da) is close to the size limit for passive paracellular transport across endothelial TJs [35]. Thus, it is sensitive to even a minor disruption to TJs [2]. The tracers were allowed to circulate for 2 h (cadaverine) or 24 h (dextran and albumin) before the animal was terminated by intracardiac perfusion of heparinised phosphate buffered saline (PBS) followed by 4% PFA. Brains were extracted and split into hemispheres which were post-fixed in 4% PFA at 4 °C for either 1 h (for pericyte assessment) or overnight (to assess BBB leakage).

### Drill injury

Buprenorphine (0.1 mg/kg) was administered to the mice. After 30 min, ketamine-xylazine (75 mg/kg-5 mg/kg) was administered. Once the animal was sleeping, the head was raised and disinfected in Betadine, and affixed to a stereotactic frame. An incision was made in the scalp, and a 1 mm diameter hole drilled in the skull, before the skin was sutured with Dafilon 5.0 sutures. The mice were monitored until waking, with extra doses of buprenorphine injected 6 h after the surgery, and the following morning.

### Immunostaining of mouse tissue

Brains were sectioned sagittally using a vibratome at either 50 μm (one-hour post-fixed tissue) or 30 μm (tissue post-fixed overnight). At least three non-adjacent sections (> 100 μm apart) were analysed per animal per condition. Sections were processed as floating sections. Sections which had been post-fixed overnight underwent antigen retrieval (incubated in 0.25% trypsin for 5 min at 37 °C, followed by 3 × 5 min washes in PBS). Sections were blocked for 2 h at room temperature on an orbital shaker in blocking solution (5% donkey serum, 0.1% triton in PBS). Sections were incubated with primary antibodies [Fibrinogen (1:5000; Dako; A0080), Glut-1 (1:500; ThermoFisher; MA5–11315), Glut-1 (1:10,000; Millipore; 07–1401), PDGFR-β (1:500; eBioscience; 14–1402-82), Aminopeptidase-N/CD13 (1:250; R&D Systems; AF2335)] in blocking solution overnight at 4 °C on a shaker. Sections were then washed in PBS (3 × 5 min) before being incubated with Alexa Fluor secondary antibodies for 4 h at room temperature on a shaker. Sections were washed in PBS (3 × 5 min), incubated with DAPI (1 min at room temperature), further washed in PBS (3 × 5 min), then mounted on slides with Dako Mounting Medium.

### Immunostaining of human tissue

Sections from paraffin blocks were cut at either 5 μm (cohort 1) or 10 μm (cohort 2) using a microtome. All samples from a cohort were processed and analysed in a single batch to minimise technical variability. Sections were deparaffinised by immersion in Toluene (2 × 2 min) and rehydrated by immersion in descending concentrations of ethanol (100, 100, 70, 50, 30%; 10 s each) before being immersed in tap water (5 min). Sections were incubated in 3% hydrogen peroxide (20 min at room temperature), followed by washing in PBS (2 × 2 min) before antigen retrieval was carried out (boiling for 10 min in citrate buffer (Vector) in a microwave). Sections were then cooled for 10 min in running tap water. Sections were blocked for 2 h at room temperature in blocking solution (for Fibrinogen: 10% goat serum, 0.1% triton in PBS; for PDGFR-β and Glut-1: 10% foetal calf serum, 0.3% triton in PBS). Sections were washed in PBS then incubated with primary antibodies [Fibrinogen (1:2500; Dako; A0080), PDGFR-β (1:50; R&D Systems; AF385), Glut-1 (1:500; Millipore; 07–1401)], Glial fibrillary acidic protein (GFAP) (1:500; Sigma-Aldrich; G-9269) in blocking solution overnight at either 4 °C (Fibrinogen) or room temperature (PDGFR-β and Glut-1). Sections were washed in PBS (3 × 5 min) then incubated with biotinylated secondary antibodies for 1 h (Fibrinogen) or 2 h (PDGFR-β and Glut-1) at room temperature. Sections were washed in PBS (3 × 2 min) then incubated with VECTASTAIN ABC reagent (Vector) for 30 min at room temperature. Sections were washed in PBS (3 × 2 min) then incubated with 3,3′-diaminobenzidine (DAB; ThermoFisher) for 5 min (Fibrinogen, Glut-1) or 30 min (PDGFR-β). Sections were washed twice in distilled water, followed by tap water (1 min), then dehydrated (10 s each in 30, 50, 70, 100, 100% ethanol; followed by 2 × 2 min in Toluene for mounting) and mounted using Eukit.

### Histological stains

#### Luxol fast blue staining

Sections were deparaffinised by immersion in toluene (2 × 2 min) and partially rehydrated in ethanol (100, 95, 95, 95%; 30 s each). Sections were incubated in Luxol Fast Blue (LFB) overnight at 37 °C. Sections were then rinsed in 3 baths of MilliQ water, then differentiated in 0.025% lithium carbonate solution (30 s) followed by 70% ethanol (20 s), then rinsed in 3 baths of distilled water. Sections were then incubated with 0.1% cresyl fast violet for 10 min at room temperature and washed in 3 baths of MilliQ water. Sections were then decoloured in 95% ethanol (3 s), dehydrated in 100% ethanol (5 s), immersed in toluene (2 × 2 min) and mounted in Eukit.

#### Haematoxylin and eosin staining

Sections were deparaffinised and rehydrated as for immunostaining. Sections were immersed in a bath of Harris haematoxylin for 30 s, then rinsed briefly in 2 baths of tap water followed by differentiation in tap water (1 min). Sections were then immersed in a bath of 1% eosin for 30 s followed by brief rinsing in 2 baths of tap water, before being dehydrated and mounted as for immunostaining.

### Imaging and analysis

#### Mouse tissue

Fluorescent images were acquired using a Leica SP8 confocal microscope, capturing a 25 μm thick stack with optical sections taken at 2 μm intervals. At least 2 fields of view were captured systematically from the most anterior and most posterior regions of the corpus callosum per section, and at least 3 sections were analysed per animal.

#### Human tissue

Bright-field images were acquired using either a Hamamatsu Nanozoomer (cohort 1) or Zeiss AxioScan (cohort 2) slide scanner, allowing us to analyse entire sections instead of a random selection of fields. Classification of WMLs was carried out by selecting regions of myelin pallor on an overview of the LFB stained section, and then examining enlargements of these regions on LFB and H&E side-by-side. WMLs around lacunes were defined as the entire area of myelin pallor on LFB around a lacune – this extended 150-600 μm from the edge of the lacune. ePVS WMLs were defined as regions of myelin pallor on LFB enriched in ePVS, which were often clustered. Where possible, fibrinogen extravasation was analysed on the entire lesion area using slide scanned images – between 1 and 20mm^2^ of tissue was analysed from each region per case.

BBB leakage was quantified using the Fiji distribution of ImageJ (open source), by automated measuring of the integrated density (encompassing both area and intensity of staining) after applying a size cut-off (mouse: > 5 μm diameter) and intensity thresholds (human: > 50, mouse: based on local mean intensity) to remove the background. Pericyte coverage in human tissue was assessed by staining for pericytes and vessels on adjacent sections, and quantified in ImageJ using a semi-automated method to calculate stained area, after manually excluding large vessels (> 10 μm) to assess only capillaries. Pericyte coverage in mouse was assessed automatically by creating a mask of the vascular area, and calculating pericyte stained area within this mask. Pericyte number in mouse tissue was quantified manually. The experimenter was blinded to the genotype throughout quantifications which were carried out manually.

### Statistics

Graphs were created and statistics calculated using GraphPad Prism. All data are shown as mean ± standard error of the mean (SEM). The number of biological replicates used in each experiment is indicated in the figure or figure legend. Significance was calculated using either a one-way or two-way ANOVA with Tukey’s post-hoc tests.

## Results

### WMLs in CADASIL patients encompass at least three categories of damage

**Fig. 1:**
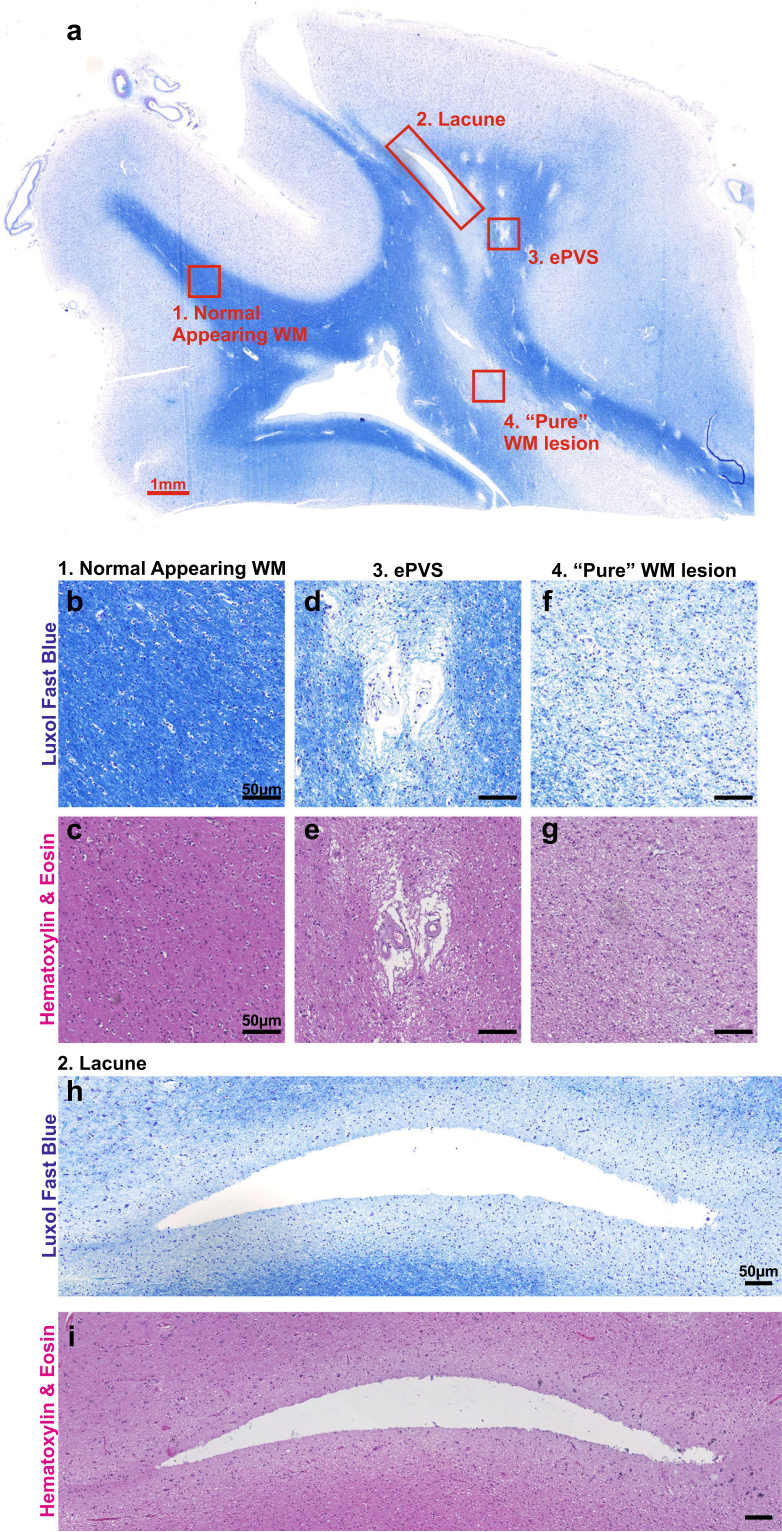
White matter lesions (WMLs) are heterogeneous in CADASIL patients. **a** Luxol Fast Blue (LFB) stained tissue section from the frontal lobe of a CADASIL patient, with different categories of white matter (WM) indicated. **b-i** LFB (**b, d, f, h**) and Haematoxylin & Eosin (**c, e, g, i**) stained images showing the regions highlighted in (**a)** in greater detail: 1. Normal appearing WM (**b-c**); 2. Lacune (**h-i**); 3. Enlarged perivascular space (ePVS) WML (**d-e**); 4. “Pure” WML (**f-g**). Scale bar: (**a**) 1 mm; (**b-i**) 50 μm

MRI studies in CADASIL patients have shown that lacunes and ePVS can occur in the same areas as WMH [6]. Therefore, before examining the BBB and pericyte integrity in post-mortem brains from patients, we first characterised the nature of WM damage. We studied 12 CADASIL patients from two separate cohorts and focused mostly on the frontal WM, as we have shown previously that the frontal lobe including WM bears the brunt of vascular pathology in CADASIL patients [8–10, 22]. We used Luxol Fast Blue (LFB) staining for myelin and Haematoxylin & Eosin (H&E) staining for general tissue structure on serial sections from paraffin embedded brains (Fig. 1). To facilitate distinction of different regions, we used a slide scanner to provide a high resolution overview of the entire section. We found that WM in CADASIL patients is more heterogeneous than is usually given consideration [20, 52]. As has been previously reported [29, 36], the myelinated U-fibres were spared and all the patients in our cohorts had some normal appearing WM (NAWM; Fig. 1a-c) as well as lesioned WM. These areas of WML were separated into three categories based on other pathological findings.

**Fig. 2:**
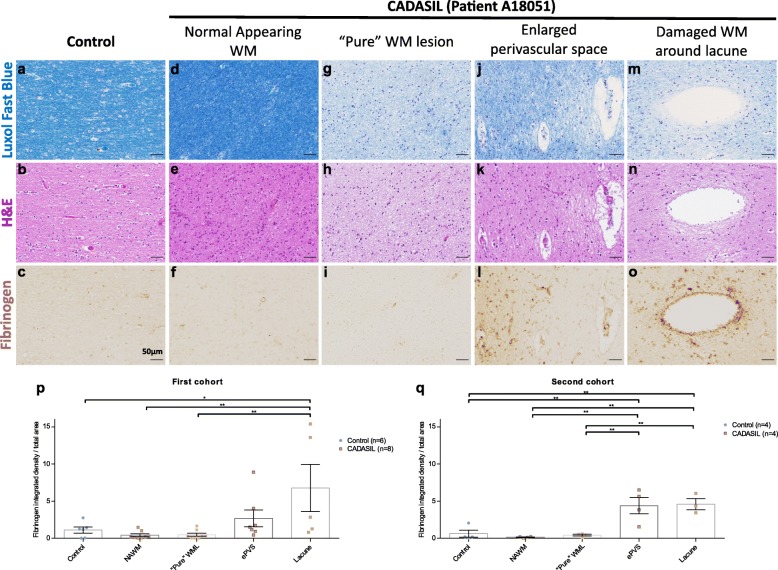
Regional heterogeneity of fibrinogen extravasation based on the category of white matter (WM) lesion (WML) in CADASIL patients. Luxol fast blue stained (**a, d, g, j, m**), Haematoxylin & Eosin (H&E) stained (**b, e, h, k, n**) and Fibrinogen immunostained (**c, f, i, l, o**) serial sections from a control brain (**a-c**), and from normal appearing WM (NAWM) (**d-f**), “pure” WML (**g-i**), enlarged perivascular space (ePVS) WML (**j-l**) and damaged WM around a lacune (**m-o**) in the brain of a CADASIL patient. Graphs show quantification of fibrinogen staining in the first (**p**) and second (**q**) cohorts of CADASIL patients. Mean ± SEM; *: *p* < 0.05, **: *p* < 0.01; one-way ANOVA with Tukey’s post-hoc tests; cohort 1: *n* = 6 (controls), 8 (patients); cohort 2: *n* = 4 (controls), 4 (patients). Scale bar: 50 μm

Lacunes on MRI are usually defined as being larger than 3 mm in diameter [48]. On histopathological sections, we detected smaller lacunes in the WM (Additional file 1: Figure S1) – as small as 300 μm in diameter (Fig. 2m), i.e. much smaller than the detection threshold of conventional 3 T MRI. Where tissue was available, reactive astrocytosis was assessed using GFAP staining and was observed around some but not all lacunes (Additional file 1: Figure S2), as expected from our previous data showing varied astrocytic responses in more recent infarcts [22]. We found that irrespective of the size of the lacune, myelin pallor was found around the border of WM lacunes, extending 150-600 μm from the edge of the lacune, that we define here as the first category of WML (Fig. 1h-i). Myelin pallor has previously been described around lacunes in ageing [29], but these are not usually analysed as a separate category of WML.

We next assessed the distribution of ePVS in the WM, and found these predominantly close to the boundary between GM and WM, at the cortico-subcortical junction (Fig. 1a). Surrounding these ePVS, we found regions of myelin pallor as the second category of WML (Fig. 1d-e). Few studies have characterised ePVS histopathologically, however an association between ePVS and WM damage has previously been shown in CADASIL [13, 51]. The patches of WM damage that we observed here around ePVS bear some similarity to lesions which have previously been described as microinfarcts, with non-uniform disorganisation of tissue structure on H&E stained sections and sizes less than 500 μm in diameter [28, 46]. This distinct patterning on H&E strongly suggests that these are a distinct type of lesion from “pure” WMLs to be described below. We have, for the present purposes, chosen not to use the terminology of microinfarcts, as this term is not well defined and implies a known pathogenic mechanism. However, in common with microinfarcts, the small size of areas of WM damage around ePVS (ePVS WMLs) precludes reliable detection on conventional MRI sequences.

The third category of WML, which we term “pure” WMLs, exhibits myelin pallor on LFB, with a homogenous structure on H&E, and does not contain any other overt pathology (Fig. 1f-g). These lesions were found exclusively in areas of deep, non-periventricular white matter (> 10 mm from the surface of the brain).

### Regional heterogeneity of white matter fibrinogen extravasation in CADASIL patients

We next evaluated the integrity of the BBB in human post-mortem tissue sections with respect to these different categories of WML. We performed DAB immunostaining for fibrinogen and quantified the integrated density of staining in different regions, incorporating both diffuse parenchymal staining and cellular uptake. We chose fibrinogen as a marker of BBB leakage due to its relevance to WMLs – in a mouse model of pericyte loss and BBB leakage, fibrinogen was suggested to be responsible for WM loss through its toxicity to oligodendrocytes [32]. We also used tissue from an acute stroke patient as a positive control to validate our immunostaining (Additional file 1: Figure S3). In the brains of our first cohort of CADASIL patients, we found strong fibrinogen staining, including cellular uptake, around the edges of lacunes (Fig. 2m-o, p) and in regions of ePVS WMLs (Fig. 2j-l, p). Morphological examination on higher magnification images suggests that both astrocytes and oligodendrocytes take up fibrinogen (Additional file 1: Figure S4). However, in regions of the brain with “pure” WMLs (Fig. 2g-i, p), without other pathology, levels of fibrinogen leakage were comparable to those seen in the normal appearing white matter (NAWM) of CADASIL patients (Fig. 2d-f, p) and age-matched controls (Fig. 2a-c, p).

To confirm this finding, we examined brains from four patients and four controls from a second cohort where we had much larger and thicker tissue sections, allowing us to better examine deep WM. In this cohort, we similarly found BBB leakage around WM lacunes and ePVS WMLs at the grey/white matter boundary, but no leakage in deeper “pure” WMLs or NAWM (Fig. 2q).

These data show that while there was evidence of BBB leakage in damaged WM in CADASIL patients, this is regionally heterogeneous. BBB leakage was present where there are ePVS or lacunes, but was not a consistent feature of WMLs in CADASIL.

### No appreciable loss of pericytes in the WM of CADASIL patients

**Fig. 3:**
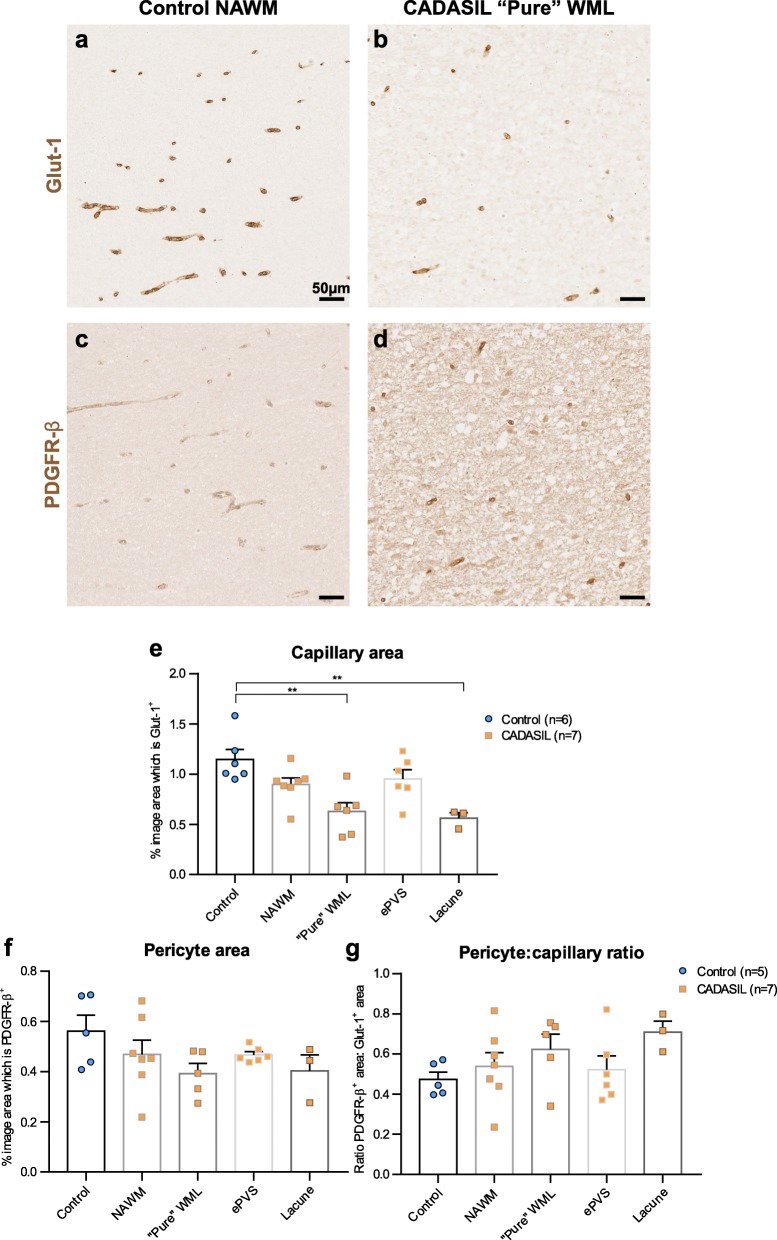
No change in pericyte coverage in CADASIL patients. Immunostaining for capillary endothelium (glut-1; **a-b**) and for pericytes (PDGFR-β; **c-d**) on serial sections from normal appearing white matter (NAWM) in a control brain (**a, c**) and a “pure” white matter lesion (WML) in a CADASIL patient brain (**b, d**). Capillary area is quantified in (**e**), pericyte area is quantified in (**f**), and the ratio of pericyte stained area to capillary stained area is quantified in (**g**). Mean ± SEM; **: p < 0.01; one-way ANOVA with Tukey’s post-hoc tests; n = 6 (controls), 7 (patients). Scale bar: 50 μm

We then assessed pericyte integrity in WM capillaries with respect to the different categories of WML. A previous study examining the frontal WM of CADASIL patients found a small increase in the PDGFR-β stained area compared to controls, but this did not distinguish between different WM areas or fully account for possible changes in capillary density [8]. We stained serial sections of tissue from CADASIL patients with Glut-1, an endothelial marker, and PDGFR-β, a marker of pericytes. We manually excluded large vessels (> 10 μm) to assess only capillaries. We found that there was a reduced capillary density in CADASIL patients compared to controls, which was significant in “pure” WMLs and around lacunes, but not in NAWM or ePVS WML (Fig. 3a-b, e). However, there was no difference in the absolute area of pericyte staining independent of capillary area (Fig. 3c-d, f), nor was there a difference in the ratio of pericyte to capillary staining (Fig. 3g).

### No BBB leakage and no pericyte loss in CADASIL mice with “pure” WML

Our findings in post-mortem tissue from human CADASIL patients show that there are different types of WML, with different BBB pathologies, although with no appreciable pericyte coverage loss. However, post-mortem tissue from aged patients represents a late stage of the disease, and thus the order of pathological changes cannot be determined. The definition of “pure” WMLs as lacking other pathology also raises the possibility of miscategorisation due to missed pathology on adjacent sections. To overcome both of these obstacles, we used a mouse model which recapitulates only the early stage of the disease.

The Tg*Notch3*^R169C^ mouse is a well characterised mouse model of CADASIL. This model overexpresses a form of the *Notch3* gene with the same mutation (R169C) which is commonly found in patients. As a control strain, we used the Tg*Notch3*^WT^ mouse, which overexpresses, through the same promoter, the wild-type form of the *Notch3* gene. This mouse model has been previously well characterised as replicating many aspects of CADASIL. Specifically, mutant mice exhibit Notch3^ECD^ accumulation around brain smooth muscle cells and pericytes as early as at 2 months of age, and develop WMLs from 6 months of age which become progressively more severe [5, 27]. Importantly however, these mice do not develop lacunes, nor do they show any signs of ePVS [27]. This allows us to study “pure” WMLs in isolation.

While previous studies have shown WMLs in the corpus callosum (CC) as a whole, we found that there is in fact a difference in the severity of WMLs across the CC. WMLs in the CC of CADASIL mice show an anterior-posterior gradient, with the WM damage much more severe in the anterior CC than the posterior CC (Additional file 1: Figure S5). Therefore, in addition to Tg*Notch3*^WT^ control animals, we were able to use the posterior CC of Tg*Notch3*^R169C^ mice as an internal control, as a region with similar vascular and axonal architecture to the anterior CC but relatively normal WM.

**Fig. 4:**
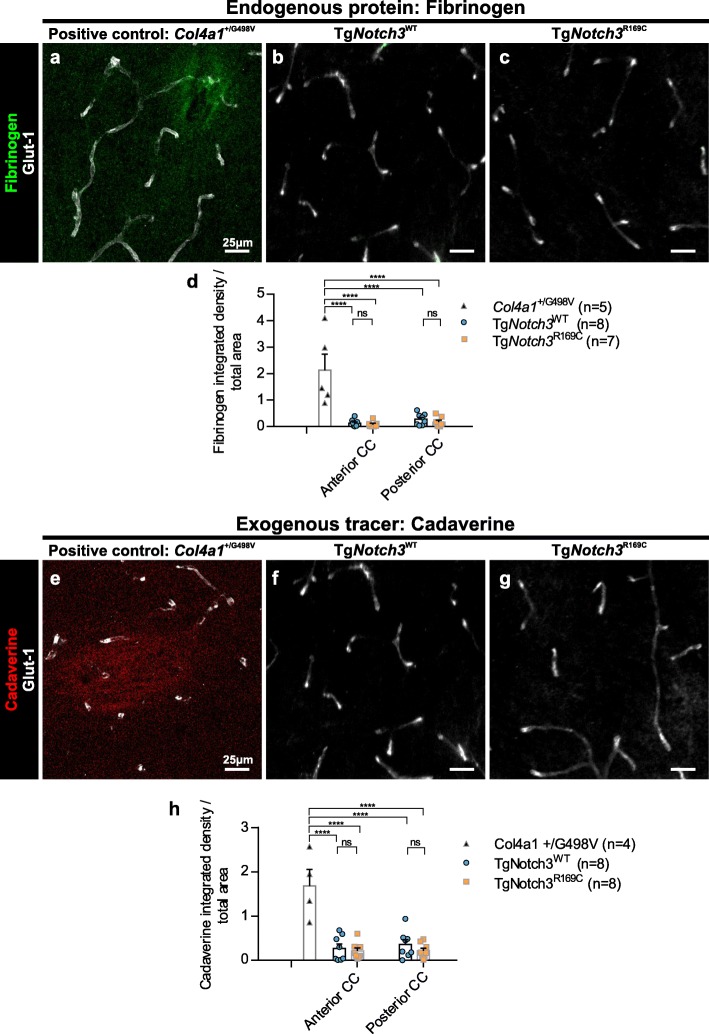
No blood brain barrier (BBB) leakage in the white matter of CADASIL mice. **a-c** Immunofluorescent images showing fibrinogen (green) and capillaries (glut-1; white) in the anterior corpus callosum (CC) of *Col4a1*^+/G498V^ (**a**), Tg*Notch3*^WT^ (**b**), and Tg*Notch3*^R169C^ (**c**) mice. Fibrinogen staining is quantified in both the anterior and posterior CC (**d**). **e-g** Fluorescent images showing injected cadaverine (red) and capillaries (glut-1; white) in the anterior CC of *Col4a1*^+/G498V^ (**e**), Tg*Notch3*^WT^ (**f**), and Tg*Notch3*^R169C^ (**g**) mice. Cadaverine leakage is quantified in both the anterior and posterior CC (**h**). Mean ± SEM; ****: *p* < 0.0001; two-way ANOVA with Tukey’s post-hoc tests; n for each group shown on graphs. Scale bar: 25 μm

We first assessed BBB integrity in the CADASIL mouse by assessing parenchymal staining of endogenous fibrinogen. As a positive control for BBB leakage to validate our techniques, we used 1 month old *Col4a1*^+/G498V^ mice, which have a mild, transient BBB leakage [42]. We found that there was no evidence of increased fibrinogen extravasation in CADASIL mice in either the anterior or posterior CC (Fig. 4a-d).

We next intravenously injected tracers of different sizes into the mice and allowed these to circulate before assessing if they had entered the brain. As the tracers used are much smaller than endogenous fibrinogen, they allow the detection of a subtler BBB defect. We first used a fluorescently conjugated cadaverine, which at 950 Da is close to the size limit of the BBB, and allowed it to circulate for 2 h. We found no evidence of cadaverine extravasation in either the anterior or posterior corpus callosum of CADASIL mice, despite clear extravasation in our *Col4a1*^+/G498V^ positive control mice (Fig. 4e-h).

While this technique allows detection of a subtle, acute BBB leakage, it may miss a slower, chronic leakage. We therefore injected a combination tracer of a labelled 70 kDa dextran and a labelled exogenous albumin (~ 70 kDa) and allowed these to circulate for 24 h. For these experiments, we used wild-type mice having undergone drill injury, which produces severe BBB leakage, as a positive control. With these tracers, we similarly found no leakage (Additional file 1: Figure S6).

**Fig. 5:**
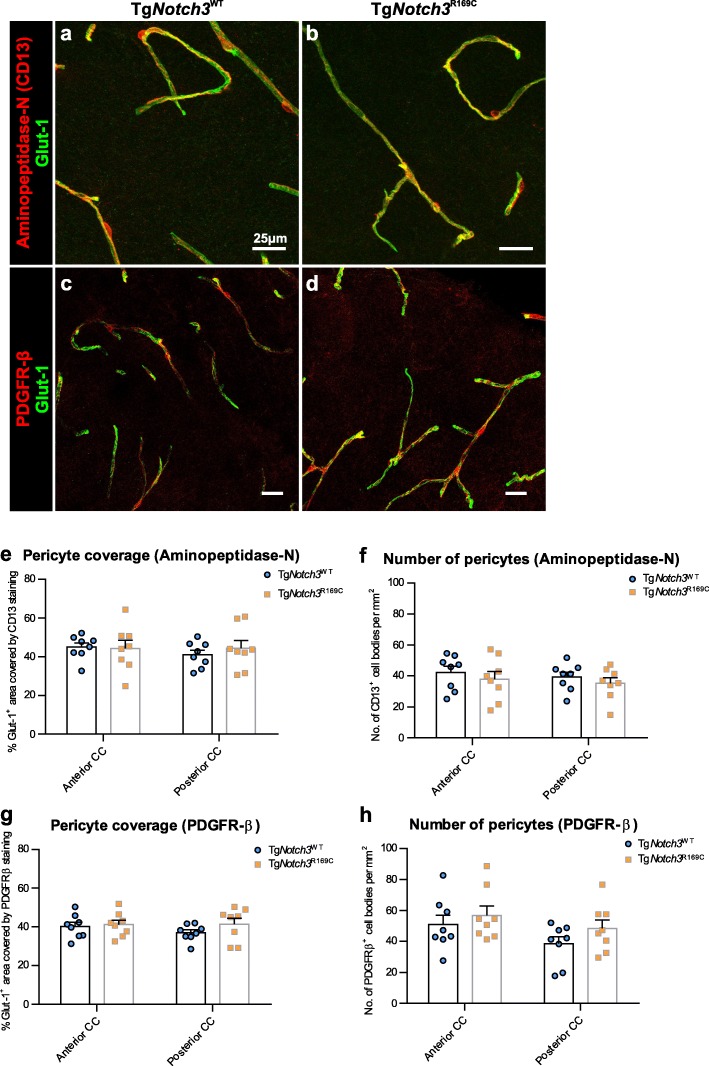
Normal pericyte coverage and number in the white matter of CADASIL mice. Immunofluorescent images showing capillaries (glut-1; green) and pericytes (**a-b**: aminopeptidase-N; **c-d**: PDGFR-β; red) in the anterior corpus callosum (CC) of Tg*Notch3*^WT^ (**a, c**) and Tg*Notch3*^R169C^ (**b, d**) mice. Quantification of pericyte coverage (**e**: aminopeptidase-N; **g**: PDGFR-β) and pericyte number (**f**: aminopeptidase-N; **h**: PDGFR-β) shows no difference in the anterior or posterior CC. Mean ± SEM; two-way ANOVA with Tukey’s post-hoc tests; *n* = 8. Scale bar: 25 μm

We also examined pericytes in the WM of CADASIL mice, where fluorescent co-staining techniques allow us to more accurately assess the pericyte coverage of capillaries. We used two different markers of pericytes, PDGFR-β and aminopeptidase-N (also referred to as CD13), to ensure reliable quantification. We found no difference in the capillary coverage of pericytes (Fig. 5a-d, e, g), or in the number of pericyte cell bodies in either the anterior or posterior CC (Fig. 5f, h).

These data together show that there is no evidence of BBB leakiness and pericyte loss in the “pure” WMLs of CADASIL mice, supporting our findings in the WM from CADASIL patients.

## Discussion

Here, we have shown that in CADASIL, a genetic paradigm of SVD: 1) WMLs are heterogeneous; 2) while there is BBB leakage in ePVS WMLs and WMLs around lacunes, the BBB is preserved in “pure” WMLs in both CADASIL patients and CADASIL mice; 3) there is no loss of pericytes in any type of WML in CADASIL patients, nor in CADASIL mice. These data together indicate that WMLs in CADASIL encompass distinct classes of WM changes and argue against the prevailing hypothesis that pericyte loss, mediated by BBB leakage, is the primary driver of all WMLs. Our results also have important implications for the biological interpretation of BBB leakage in CADASIL WM assessed with conventional 3 T MRI, since the large voxel size (~ 1 mm^3^) precludes reliable separation of these distinct categories of WMLs.

Given our experimental observations presented above, we propose a new classification of different types of WMLs. This is especially important when considering comparisons to MRI studies that usually define different types of WMH based on localisation in different brain regions or different depths within the brain, but not based on the presence of other pathological features within WMLs [44]. Our findings that “pure” WMLs are exclusively located in deep WM, while ePVS WMLs tend to be distributed in subcortical WM at the GM/WM boundary suggests that classification of WMLs based on depth could provide some overlap with the categories we have defined. However, the definitions of different regions of depth within the brain differ in MRI studies, and the deep and subcortical WM are often combined into a single category to be compared against periventricular WM [44], which we unfortunately did not have access to for this study. Moreover, the small size of many ePVS, and even some lacunes, can make them invisible on conventional 3 T MRI sequences, which often cannot detect pathology smaller than 1 mm in diameter. Thus, it is possible that WMLs associated with these small ePVS or lacunes show up as WMHs, indistinguishable from “pure” WMLs. Studies on the BBB in living patients may therefore misinterpret evidence of BBB leakage within WMHs as suggesting a BBB related mechanism for all WMLs, when in fact this may only apply to a subset of these lesions. More advanced quantitative MRI techniques that provide biophysical parametric measurements, sometimes called “*in vivo* histology”, may enable discrimination of these based on different water and lipid content [13, 19], however these methods are not in common usage.

Despite these limitations of conventional MRI sequences, the possibility that WM in CADASIL encompasses distinct classes of lesions has been suggested by recent MRI studies [13, 17]. Lesions in the WM of anterior temporal poles and superior frontal gyri, which are locations highly suggestive of CADASIL, have higher T1 and T2* relaxation times and are associated with better cognitive and clinical outcomes [13, 17]. Importantly, high resolution 7 T structural MRI suggested a close spatial relationship between this class of WMLs and ePVS at the cortico-subcortical junction [13], as we have shown here on post-mortem tissue. This is further evidenced by our previous histopathological study suggesting that WMH seen on MRI in the temporal poles of CADASIL patients are predominantly explained by ePVS and associated WM degeneration [51].

We confirmed the lack of BBB leakage in “pure” WMLs in a mouse model of CADASIL, which allowed us to look at an earlier stage of the disease than is possible with human post-mortem tissue, such that all WMLs are “pure” without ePVS or lacunes. While BBB defects have previously been suggested in this model, this was only examined in the cortex and only by studying endogenous blood-borne proteins [21]. While the examination of endogenous proteins is a more biologically relevant assay of BBB leakage, using this method alone is not always reliable as these proteins may have entered the parenchyma by other routes. It is for this reason that, in this study, we examined both endogenous blood-borne proteins and multiple different exogenous injected tracers. Examination of the cortex is less relevant to the mechanisms of WMLs. Moreover, quantification of a diffuse fluorescent staining in the cortex of aged animals also introduces the possibility of unreliable measurements due to the presence of lipofuscin. Lipofuscin appears as autofluorescent granules around the nuclei of neurons where lysosomes accumulate with age, and can be difficult to distinguish from real fluorescent staining due to its broad fluorescence emission spectrum [30]. Assuming that increased fluorescent staining for blood-borne proteins observed in this previous study [21] was real, this could be caused by differences in the environments in which the animals are housed. It has been shown that differences in gut microbiota can alter the permeability of the BBB [39]. This, however, does not detract from the fact that the mice in our colony did not display any signs of BBB leakage in the WM but still developed severe WMLs, suggesting that BBB leakage cannot be the primary cause of these lesions.

We have used a range of techniques to show that there is no generalised BBB leakage in CADASIL WM, however our study does have some limitations. One of these is the variability of immunohistochemical staining techniques between different runs, especially when quantifying a diffuse signal. We minimised this variability by immunostaining all human samples from a cohort in a single batch, and analysing each cohort separately. This batch to batch variability, along with the different brain bank origins and different imaging platforms used to accommodate larger slides, explains the difference in values of fibrinogen integrated density between the first and second human cohorts. Another limitation of our study is that the techniques used, notably the tracer experiments in the mouse, primarily probed the integrity of TJs of the BBB, and did not fully exclude the possibility of a BBB leakage through the transcellular pathway of increased vesicular transport in endothelial cells. This possibility can only be fully excluded by analysis of electron micrographs following injection of biotin or horse radish peroxidase to mark vesicles. However, pericyte loss causes both a loss of TJs and an increase in vesicular transport [2, 12], thus we would expect to see extravasation of tracers were the hypothesis posited in the introduction correct. Furthermore, endogenous fibrinogen has been shown to cross the BBB by the transcellular pathway as well as across TJs [33].

Distinguishing these different types of WMLs is important, as we show here that ePVS WMLs and WMLs around lacunes display BBB leakage, while “pure” WMLs do not, indicating that new avenues need to be explored to understand the mechanisms behind this type of lesion. One possibility is reduced oxygen delivery to these regions, leading to the death of oligodendrocytes which are especially sensitive to hypoxia [15]. The reduced capillary density which we see in these lesions supports the notion of reduced blood supply causing WMLs, however it is equally likely that this reduced capillary density may be a consequence of the reduced metabolic demands in a demyelinated lesion. Reduced oxygen supply to these regions could also be due to the impaired cerebrovascular reactivity which has been reported in the WM of CADASIL patients [3, 40]. This impaired cerebrovascular reactivity could also lead to a failure of adequate drainage of interstitial fluid from the WM along cerebral arteries, causing a build-up of substances toxic to cells of the WM [49]. Another possibility is that these lesions are caused by aberrant signalling from the vasculature to oligodendroglia, or a lack of trophic support for myelin. Studies in models of sporadic SVD have shown that signalling from dysfunctional endothelial cells can cause WM vulnerability [41]. Recently, it has been shown that pericytes secrete factors which provide trophic support to neurons, and these factors may also play a similar role for myelin [34]. In CADASIL mice, it has been shown that vitronectin accumulation around blood vessels plays an important role in the development of WMLs [5], and it may be that this and other factors signal to oligodendrocytes and alter the stability of myelin.

## Conclusions

Here, we have demonstrated that BBB leakage is not a consistent feature of WMLs in CADASIL, and is only present where there are lacunes or ePVS. Thus, it is unlikely to be the primary mechanism of pure WM damage in this disease. These findings may also have implications for sporadic SVD and other dementias in which WMLs are present [23].

## Supplementary information

**Additional file 1.** Figures S1-S6 and Tables S1-S2.

## Data Availability

The authors declare that the data supporting the findings of this study are available within the article and its additional files. Full datasets and materials are available upon reasonable request.

## Abbreviations

BBB: Blood brain barrier
CADASIL: Cerebral autosomal dominant arteriopathy with subcortical infarcts and leukoencephalopathy
CC: Corpus callosum
ePVS: Enlarged perivascular spaces
GM: Grey matter
H&E: Haematoxylin and eosin
LFB: Luxol fast blue
MRI: Magnetic resonance imaging
NAWM: Normal appearing white matter
SEM: Standard error of the mean
SVD: Small vessel disease
TJ: Tight junction
WM: White matter
WMH: White matter hyperintensities
WML: White matter lesion
