# Supplementary material for: Blood brain barrier leakage is not a consistent feature of white matter lesions in CADASIL
Source: Acta Neuropathol Commun. 2019 Nov 21;7:187. doi: 10.1186/s40478-019-0844-x (PMC6873485; doi:10.1186/s40478-019-0844-x)
Supplement: Supplementary file 1 — Additional file 1. Figures S1-S6 and Tables S1-S2. [file 40478_2019_844_MOESM1_ESM.pdf]

**Figure S1**

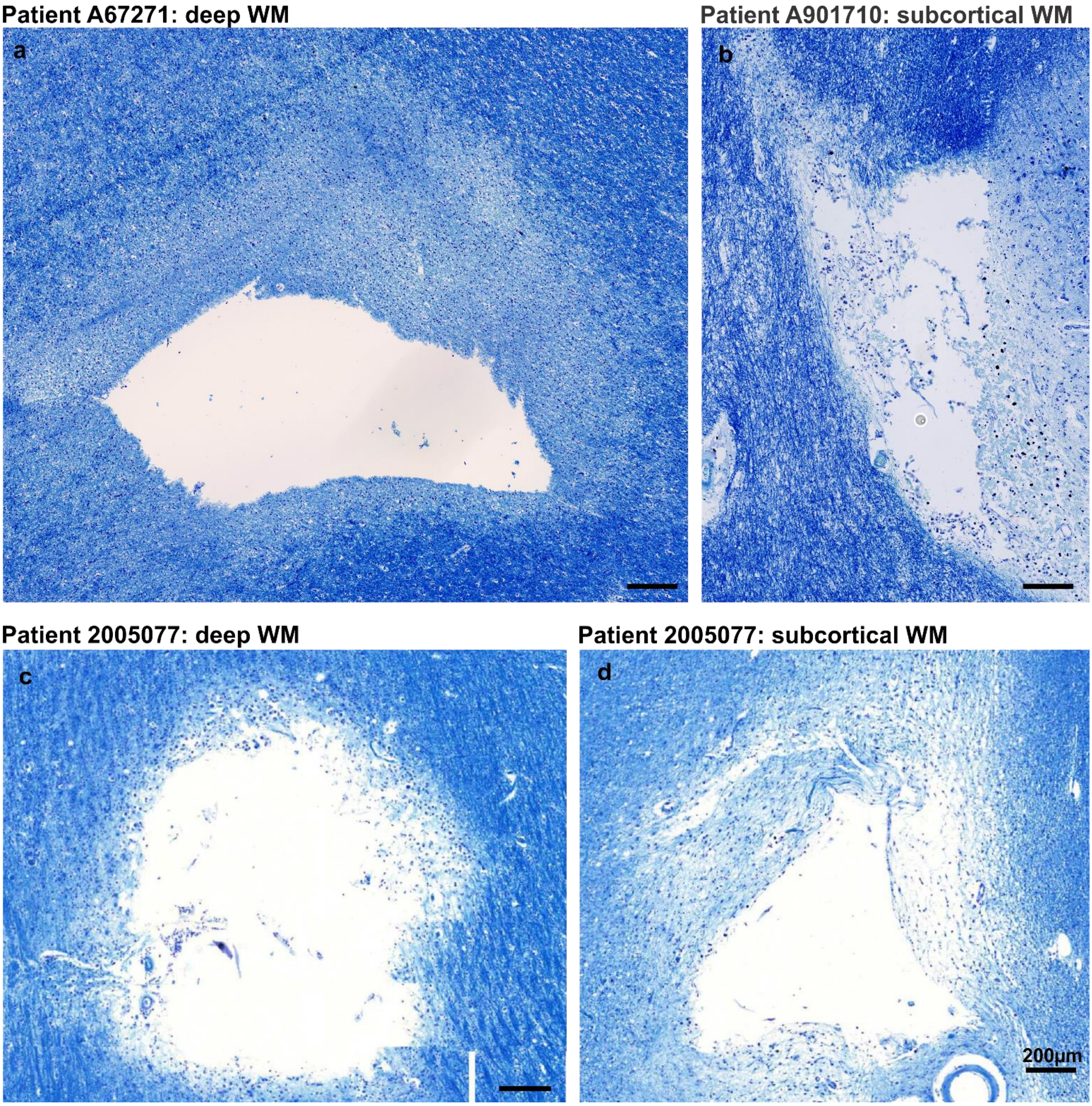


**Figure S1** Examples of lacunes smaller than the MRI definition of 3 mm diameter. Luxol fast blue stained sections from the white matter of 3 CADASIL patients, with lacunes in the deep (**a, c**) and subcortical (**b, d**) white matter ranging in diameter from 800 µm to 1.5 mm. These are all smaller than the MRI definition of a lacune being >3 mm in diameter [40]. All of these lacunes show a collar of myelin pallor, the width of which is unrelated to the diameter of the lacune. Scale bar: 200 µm.

**Figure S2**

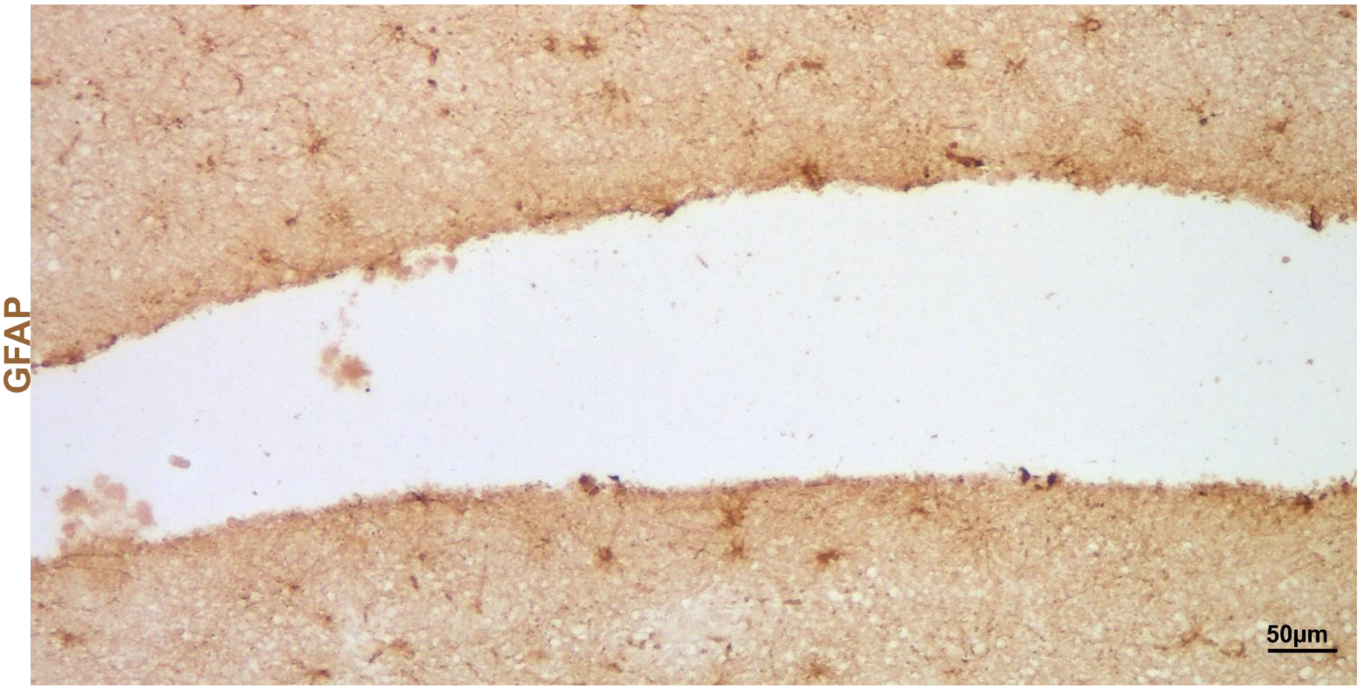


**Figure S2** Astrocytosis around a lacune. GFAP immunostaining around the lacune shown in **Fig. 1 h-i**, showing astrocytosis around the lacune.

**Figure S3**

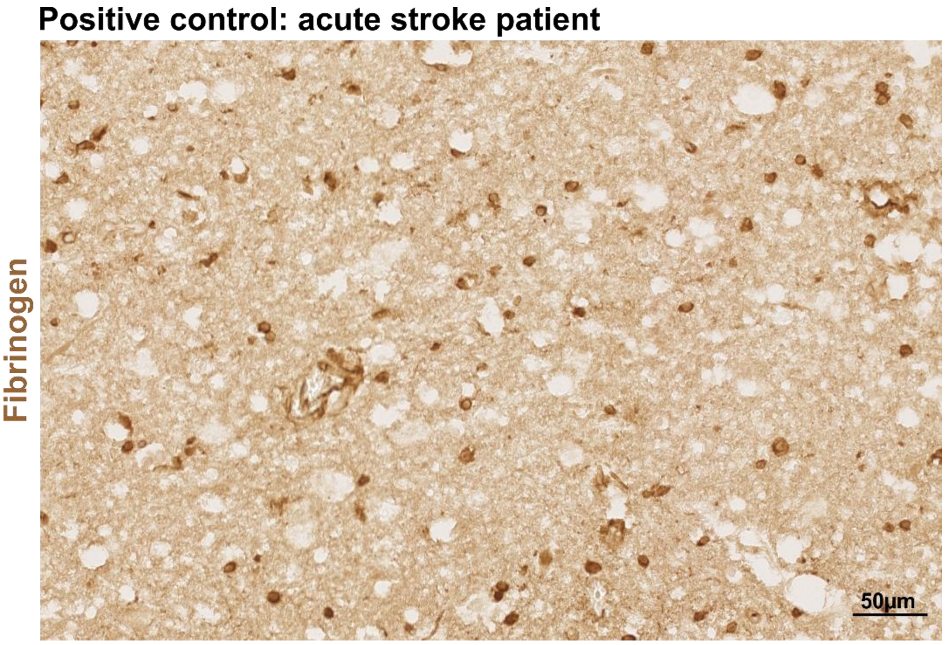


**Figure S3** Fibrinogen staining in an acute stroke patient, as a positive control to validate our immunohistochemical techniques.

**Figure S4**

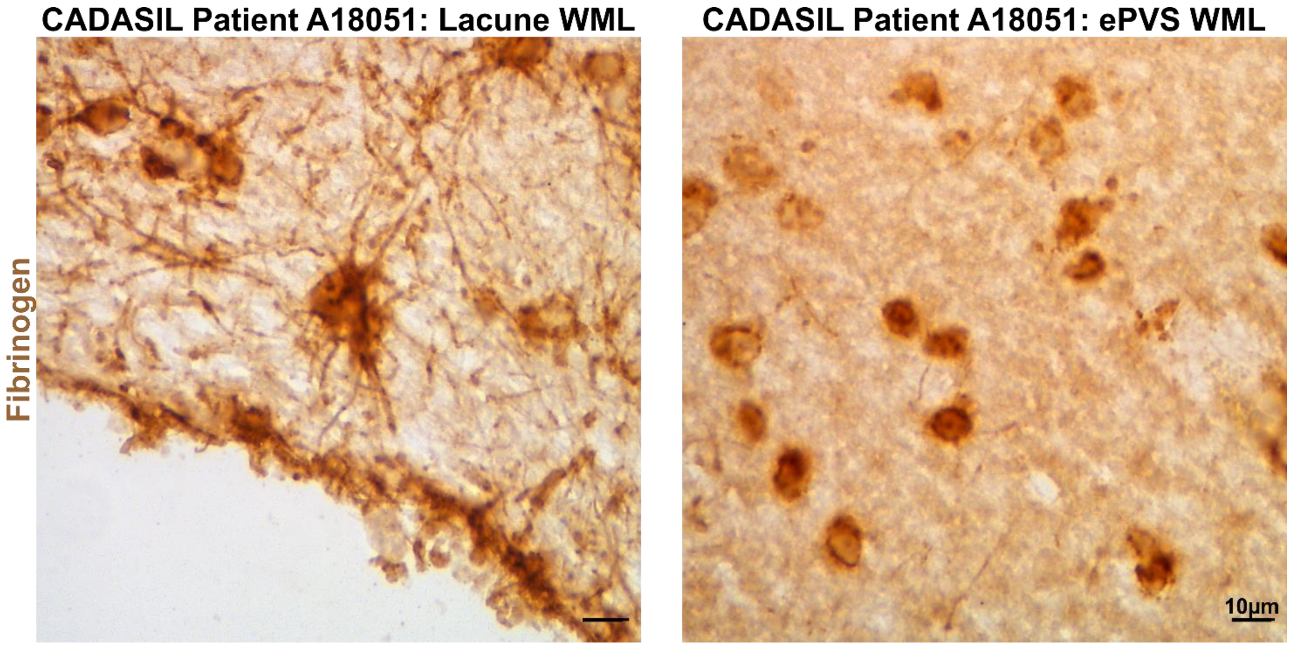


**Figure S4** High magnification images showing fibrinogen immunostaining in the lacune WML and ePVS WML shown in **Fig. 2**. The morphology of the cells stained with fibrinogen suggests that both astrocytes and oligodendrocytes take up fibrinogen.

**Figure S5**

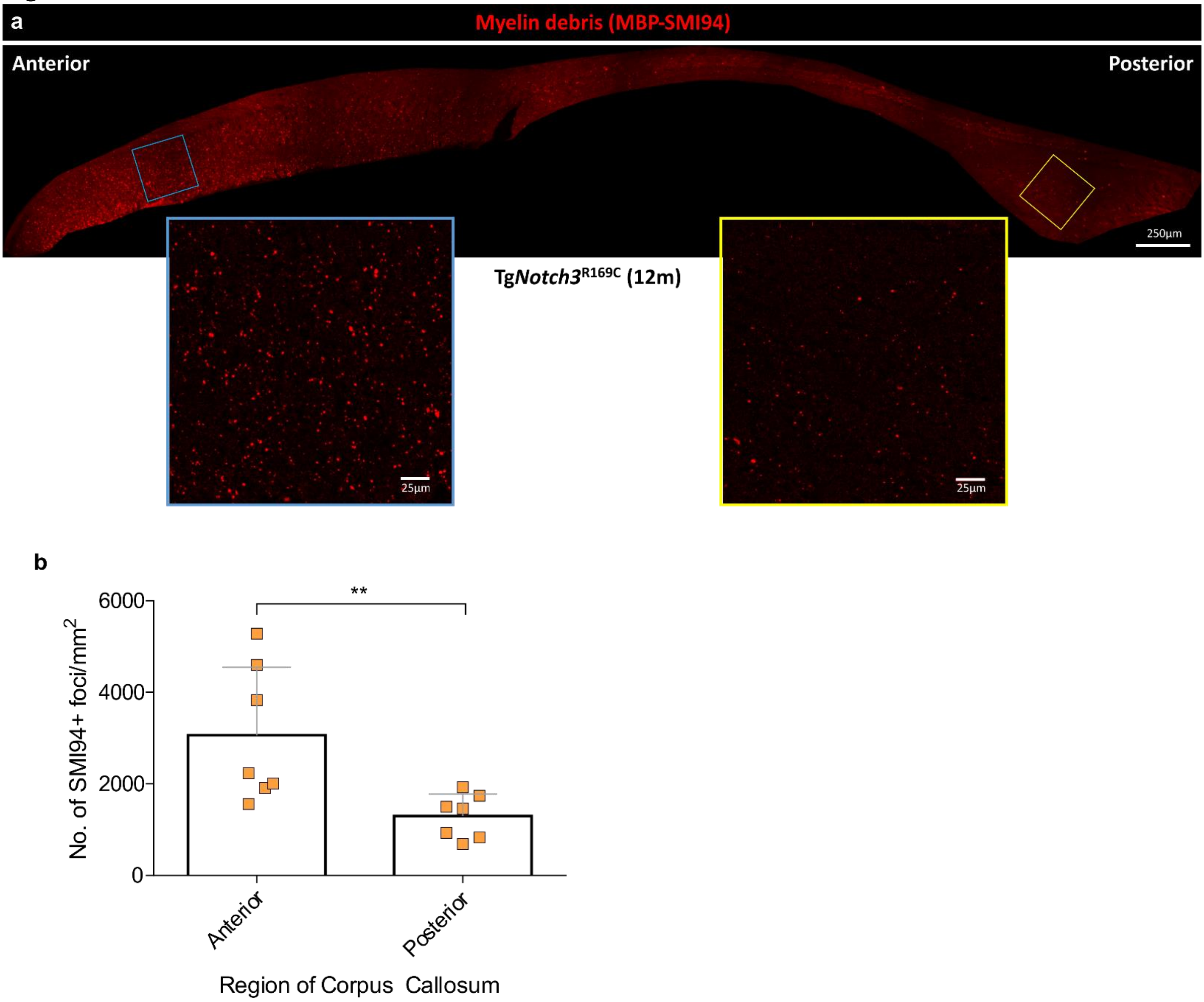


**Figure S5** White matter lesions are more severe in the anterior corpus callosum than in the posterior corpus callosum in CADASIL mice. **a** Overview of the corpus callosum from a Tg*Notch3*$^{R169C}$ mouse immunostained with an antibody against damaged/decompacted myelin (MBP-SMI94; red). With this antibody, increased foci of staining indicate greater myelin damage and a more severe lesion. Enlargements of areas from the anterior (blue box) and posterior (yellow box) regions are shown. **b** Quantification of the number of foci of myelin debris in 12 month old Tg*Notch3*$^{R169C}$ mice shows more myelin damage in the anterior corpus callosum than in the posterior corpus callosum. Mean ± SEM. **: p<0.01; paired t-test; n=7. Scale bar: 250 µm (overview); 25 µm (enlarged areas).

**Figure S6**

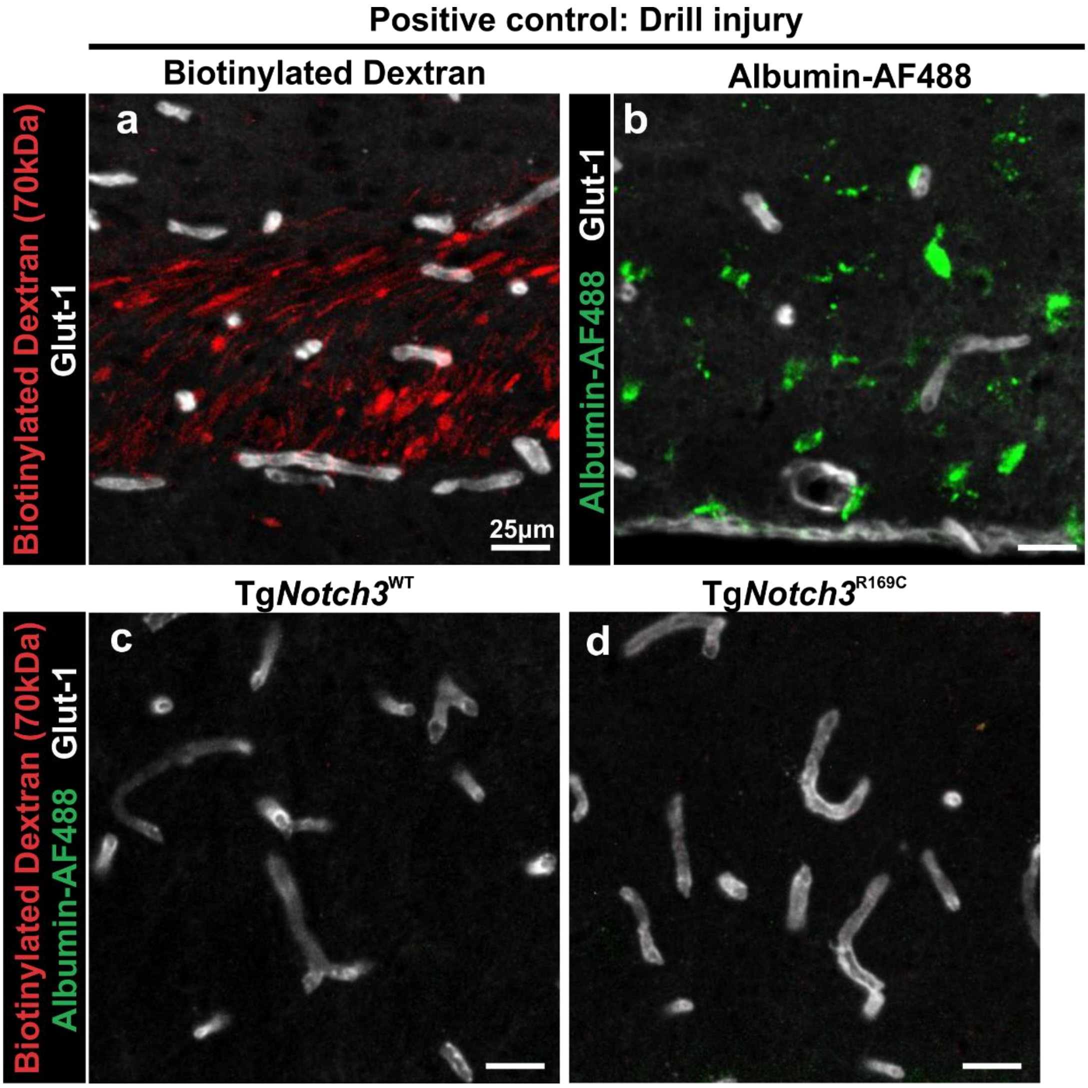


**Figure S6** No leakage of large tracers through the blood brain barrier (BBB) of CADASIL mice. Fluorescent images showing capillaries (glut-1; white), as well as an injected 70kDa dextran (red) and injected labelled albumin (70kDa; green) extravasated in control mice having undergone drill injury (**a-b**, different fields of view), while there is no leakage in Tg*Notch3*$^{WT}$ mice (**c**) or Tg*Notch3*$^{R169C}$ mice (**d**). Scale bar: 25 µm.

**Table S1**

| Controls | | | | |
|---|---|---|---|---|
| **First cohort** | | | | |
| **ID** | **Gender** | **Age (years)** | **Post-mortem delay (hours)** | **Brain region** |
| **A953196** | M | 48 | 25 | Temporal lobe |
| **A98203** | F | 63 | 10 | Frontal lobe |
| **5875** | F | 47 | 13 | Frontal lobe |
| **82A** | M | 52 | 13 | Frontal lobe |
| **A962432** | M | 84 | 32 | Frontal lobe |
| **SD008/17-001.29824** | F | 71 | 96 | Frontal lobe |
| **Second Cohort** | | | | |
| **ID** | **Gender** | **Age (years)** | **Post-mortem delay (hours)** | |
| **20090118** | M | 55 | <48 | Frontal lobe |
| **20060093** | F | 78 | 23 | Frontal lobe |
| **20080005** | F | 59 | <48 | Frontal lobe |
| **20060081** | F | 58 | 39 | Frontal lobe |

**Table S1. Details of human control cases from which post-mortem brain tissue was obtained.**

**Table S2**

| Patients | | | | | | | | |
|---|---|---|---|---|---|---|---|---|
| **First cohort** | | | | | | | | |
| **ID** | **Gender** | **Age (years)** | **Mutation** | **Post-mortem delay (hours)** | **"Pure" WML** | **ePVS** | **Lacune** | **Brain region** |
| **A67271** | F | 60 | R153C | 24 | ✓ | ✓ | ✓ | Frontal lobe |
| **A0017989** | F | 70 | C1261R | 12 | ✓ | ✓ | ✓ | Frontal lobe |
| **A18051** | F | 70 | R110C | 4 | ✓ | ✓ | ✓ | Frontal lobe |
| **110A** | M | 49 | R169C | 4 | ✓ | ✓ | - | Frontal lobe |
| **A8803** | M | 54 | R90C | 23 | ✓ | ✓ | - | Occipital lobe |
| **A18-826** | M | 77 | C428S | 24 | ✓ | ✓ | - | Frontal lobe |
| **A901710** | M | 64 | R153C | 20 | ✓ | - | ✓ | Frontal lobe |
| **N93-301** | F | 59 | R133C | 24 | ✓ | ✓ | ✓ | Frontal lobe |
| **Second Cohort** | | | | | | | | |
| **ID** | **Gender** | **Age (years)** | **Mutation** | **Post-mortem delay (hours)** | **"Pure" WML** | **ePVS** | **Lacune** | |
| **2006056** | F | 68 | R133C | 25 | ✓ | ✓ | - | Frontal lobe |
| **2010074** | M | 68 | R153C | 12 | ✓ | ✓ | ✓ | Frontal lobe |
| **2005077** | M | 55 | R558C | 24 | ✓ | ✓ | ✓ | Frontal lobe |
| **20001OX** | F | 52 | R133C | 24 | ✓ | ✓ | ✓ | Frontal lobe |

**Table S2. Details of human CADASIL patients from which post-mortem brain tissue was obtained.**
